# The effectiveness of the information‐motivation model and domestic brushing with a hypochlorite‐based formula on peri‐implant mucositis: A randomized clinical study

**DOI:** 10.1002/cre2.487

**Published:** 2021-10-22

**Authors:** Saverio Cosola, Giacomo Oldoini, Enrica Giammarinaro, Ugo Covani, Annamaria Genovesi, Simone Marconcini

**Affiliations:** ^1^ Department of Stomatology Tuscan Stomatologic Institute, Foundation for Continuing Education and Dental Research Camaiore (LU) Italy; ^2^ Department of Stomatology, Faculty of Medicine and Dentistry University of Valencia Valencia Spain; ^3^ Department of Stomatology Saint Camillus International University of Health Sciences (UniCamillus) Rome Italy

**Keywords:** chlorhexidine, full‐mouth disinfection, nitradine, PerioTabs®

## Abstract

**Objective:**

Management of mucositis is essential for the long‐term maintenance of dental implants. This study determined the efficacy, in terms of clinical parameters, of an adjunctive domiciliary agent paired with non‐surgical periodontal therapy (NSPT) for patients with peri‐implant mucositis.

**Materials and methods:**

Patients involved in a periodontal maintenance program were randomly distributed to the domestic use of a chlorhexidine toothpaste and mouthwash (control) or a hypochlorite‐based formula brushing solution (test) after diagnosis of peri‐implant mucositis. A modified approach towards NSPTwas performed after 10 days of domestic use of the assigned maintenance product in both groups. Clinical and patient‐related outcomes were recorded during a 90‐day follow‐up period.

**Results:**

Forty patients completed the three‐month study (20 patients per group). Both groups showed relevant clinical and patient outcome improvements after the NSPT (T2) and between T1 and T2 (*p* < 0 0.01), except for PPD. For the test group, the clinical improvement was significantly greater than that for the control group at the seventh‐day evaluation (T_1_) in the gingival index (0–3) and FMBS (%). Favorable outcomes were maintained during the entire follow‐up period.

**Conclusion:**

The present study showed that the modified NSPT paired with the domestic use of nitradine‐based formula helps resolve peri‐implant mucositis and that nitradine might represent an alternative to chlorhexidine in these cases.

**Clinical relevance:**

The gold standard for nonsurgical maintenance is full‐mouth disinfection. A previous decontamination of the oral cavity with chlorhexidine or nitradine domiciliary for 10 days could reduce plaque and inflammation, resulting in a painless operative session. This protocol may help reduce airborne contamination and the risk of cross‐infection, and during the pandemic, the protocol is safer for clinicians. In the same clinical cases, nitradine may be more efficient than chlorhexidine, and the former has no side effects such as discolouration.

## 1 INTRODUCTION

It is well known that the presence of bacterial plaque is associated with the development of gingival and periodontal diseases, including periodontitis and dental implant infections. Peri‐implant diseases are a group of infectious‐inflammatory pathologies that affect hard and soft tissues surrounding dental implants that, if not treated, can lead to the implant's loss of osteointegration and the need for its removal (Cosgarea et al., 2019).

The diagnostic criteria for peri‐implant mucositis and peri‐implantitis have recently been redefined at the World Workshop of Periodontal and Peri‐implant Disease (2018). According to this classification, peri‐implant conditions are grouped as: peri‐implant health, peri‐implant mucositis, peri‐implantitis, and soft‐ and hard‐tissue deficiencies (Berglundh et al., 2018). The main causative factor for peri‐implantitis is the presence of bacteria organized as biofilm. However, the clinical manifestation might depend on other local (severe periodontal disease, position of the implant, prosthetic malocclusion, or erroneous use of biomaterials) and systemic (severe systemic illnesses or personal habits such as smoking, alcohol consumption, or drug abuse) factors (Canullo et al., 2017). Peri‐implantitis is often preceded by untreated peri‐implant mucositis and is associated with intense bleeding and eventual suppuration. The main diagnostic criterion that differentiates peri‐implantitis from peri‐mucositis is progressive marginal bone loss when compared with the baseline radiography (Mombelli, 2018).

Therefore, the development of appropriate protocols for the prevention of infectious implant disorders through efficient oral hygiene maintenance is crucial, as early diagnosis and successful treatment of peri‐implant mucositis could reverse the disorder and may stop further progression of the disease towards peri‐implantitis (Coli & Sennerby, 2019).

The first step in preventing periodontal diseases is non‐surgical periodontal therapy (NSPT), which consists of scaling and root planning by manual and ultrasonic instruments to remove supra‐gingival and subgingival plaque. NSPT is usually performed following two different protocols, “quadrant” or “one‐stage” full‐mouth disinfection (FMD) (Van der Weijden et al., 2019). One‐stage FMD has the advantage of avoiding any potential bacterial translocation from a treated site to an untreated site (Quirynen et al., 2006).

The modified full‐mouth disinfection (MFMD) protocol was introduced by Genovesi and colleagues in 2014, to spare a patient from an unpleasant one‐stage sitting, by means of preliminary domestic use of chlorhexidine and sonic toothbrush (Genovesi et al., 2014). Without any initial treatment, patients who undergo complete FMD at the first appointment may be exposed to the risk of a consequent systemic inflammation, bacteraemia, pain, and discomfort during plaque removal (Graziani et al., 2015). Meanwhile, the MFMD approach is well tolerated by patients and is associated with less bleeding and less need for anesthesia when compared with standard FMD (Marconcini et al., 2019).

A preoperative rinse with an antimicrobial agent such as chlorhexidine is recommended in surgical periodontal treatment protocols (Quirynen et al., 2000; Zhao et al., 2020). Nevertheless, chlorhexidine has several side effects; thus, new decontaminating products such as NitrAdine® are emerging in dental practice (Slot et al., 2014).

NitrAdine® comes in an easy‐to‐use brushing solution (PerioTabs®), and it is a composite formula (Silva‐Lovato et al., 2010). Its anti‐biofilm mode of action is based on a combination of surfactant‐induced protein denaturation and slow release of a non‐toxic concentration of hypochlorite (0.02%) effective versus micro‐organisms including bacteria, fungi, and viruses (Glass et al., 2004). PerioTabs® is classified as medical device class I in the European Union (Patent pending EP15798353.7 and US 15/526,247) with no side effects reported (no teeth discolouration, corrosion, or burning sensations), and it is used for decontamination purposes in different oral care‐related issues (Vento‐Zahra et al., 2011). This antimicrobial product has been proven useful in different clinical scenarios (Coenye et al., 2008; Goguta et al., 2021).

This randomized controlled clinical study evaluated the clinical efficacy of full‐mouth decontamination paired with 10‐day brushing with the PerioTabs® brushing solution versus the use of chlorhexidine toothpaste combined with chlorhexidine mouthwash. The null hypothesis was that no significant clinical differences exist between chlorhexidine and PerioTabs® when combined with full‐mouth decontamination.

## 2 MATERIALS AND METHODS

### 2.1 Study design

This study was a randomized controlled clinical trial with a three‐month follow‐up period. Eligible patients were enrolled from those attending the Tuscan Stomatologic Institute (Lido di Camaiore, Italy)—for regular implant maintenance protocols—that eventually presented with peri‐implant mucositis. Ethical approval was granted by Saint Camillus International University of Health Sciences (UniCamillus, Rome), number 6/2020 (Protocol: “Studio clinico sull'efficacia della motivazione all'igiene orale domiciliare mediante un dispositivo medico in pazienti con mucosite perimplantare”). All patients signed written informed consent at the time of enrolment in the study following the 2008 Helsinki Declaration, updated in 2013.

### 2.2 Inclusion and exclusion criteria

The main inclusion criterion was the presence of ≥1 endosseous dental implant with clinical and radiographic signs of peri‐implantitis and/or peri‐implant mucositis around one or more implants per patient. Peri‐implant mucositis was identified through a visual examination of edema and inflammation, with peri‐implant probing pocket depth (PPD) ≥ 5 mm and the presence of suppuration or bleeding upon probing. Peri‐implantitis was identified through observation of bleeding and/or suppuration upon probing, combined with a PPD ≥5 mm and bone loss ≥2 mm from the marginal bone level (MBL) at implant loading (Dietrich et al., 2019).

The following inclusion criteria were adopted: implant function time ≥ 1 year, patients aged 18 years or older, patients exhibiting good general health with no systemic disorders that might affect implant health (such as uncontrolled diabetes, cardiovascular events, or immunodeficiency), patients able to comply with the study protocol, and willingness to adhere to the hygiene instructions. Exclusion criteria were alcohol or drug abuse; regular drug use of bisphosphonates; use of non‐steroidal or steroidal anti‐inflammatory medication, daily antacid therapy, selective serotonin reuptake inhibitors, and other therapies that might affect implant health; smoking more than 10 cigarettes; pregnancy; lactation; previous periodontitis treatment within the last 6 months; radiotherapy to the head or neck; and current chemotherapy. Subjects exhibiting at least one of these criteria were excluded from the present study. Full medical and dental histories were recorded, along with an oral examination.

### 2.3 Main variables

The main variables are as follows:

- Gingival index (GI): average full mouth score (ranging from 0 to 3);
- Mean pocket probing depth (PPD): average full mouth score (expressed in mm);
- Recession (Rec): average full mouth score (expressed in mm);
- Full‐mouth bleeding score (FMBS): average full mouth score (expressed in %);
- Modified plaque index (MPI) by Mombelli (1987) for dental implant scales as follows: 0 = no plaque, 1 = plaque at the cervical margin difficult to be seen, 2 = plaque can be seen by the naked eye, 3 = abundance of soft matter;
- Modified sulcus bleeding index (MSBI) for dental implant scales as follows: 0 = no bleeding when periodontal probe is passed along the gingival margin, 1 = isolated bleeding spots visible, 2 = blood forms a confluent red line on the gingival margin, 3 = heavy or profuse bleeding;
- MBL around dental implants by radiographic assessment;
- Pain reported by patients, measured via a visual analogic scale (VAS) with scores ranging from 1 to 10; and
- Patients' reported outcome: oral health‐related quality of life (OHrQoL), with scores ranging from 0 (good impact) to 5 (negative impact), measured at T0 and T1.

Main intervention: *Full‐mouth decontamination*

Full‐mouth decontamination was performed according to the modified approach by Genovesi et al. (2014), which consisted of two appointments:

- Baseline: included instruction and motivation with extensive visual explanation of the correct use of antimicrobial domestic products and devices, and
- Professional treatment: included one‐stage FMD using manual and sonic instruments.

### 2.4 Randomization and allocation

The patients were randomly allocated to one of two possible treatment groups (20 patients per group) by drawing a card. Sample size estimation was performed according to previously published literature to achieve a significant difference in the longitudinal analysis of implant clinical parameters. The sample size was calculated using a statistical software (i.e., Stata 12.0, StataCorp LLC 4905 Lakeway Drive College Station, Texas 77845‐4512 USA), based on the results of clinical periodontal parameters in previous studies (Goguta et al., 2021; Marconcini et al., 2019).

#### 2.4.1 Group 1 or test group (N group)

At baseline, the patients were given instructions for the domestic use of PerioTabs® for daily, 2‐min gum and tooth brushing during a 10‐consecutive‐day preparatory period. They were provided a PerioTabs® box containing 10 small effervescent tablets (one per day). Each evening, the patients dissolved a tablet in the provided container filled with 15 mL of lukewarm water to create a gum and tooth brushing solution. The brushing process was carried out with either a regular or soft bristle toothbrush by immersing it in the solution while the tablet was dissolving. The NitrAdine® formula contains the following substances: citric acid, sodium lauryl sulfate, lactose monohydrate, sodium bicarbonate, sodium chloride, potassium monopersulfate, sodium carbonate, peppermint flavor, and polyvinylpyrrolidone (Vento‐Zahra et al., 2011). The next morning, the patients brushed their teeth and gums with water only, using a manual toothbrush. After 1 week, full‐mouth decontamination was performed using ultrasonic devices on each quadrant, with particular care paid to the inflamed pockets around the teeth and implants.

#### 2.4.2 Group 2 or control group (C group)

At baseline, the patients were given instructions for the domestic use of a manual toothbrush with chlorhexidine 0.12% toothpaste for 10 days of tooth brushing twice daily (morning and evening). In addition, the patients used a chlorhexidine 0.2% mouth rinse every evening for 10 days. After 1 week, full‐mouth decontamination was performed in the same way as in Group 1.

#### 2.4.3 Timeline

The study comprised one baseline and four follow‐up visits defined as follows, as reported in Table 1:

**Table 1:** Timeline of the study

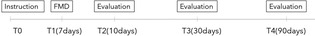

T_0_ = baseline and oral hygiene instruction session;

T_1_ = 7 days after the oral hygiene instruction session, full‐mouth debridement;

T_2_ = 10 days after the oral hygiene instruction session and 3 days after full‐mouth debridement;

T_3_ = 30 days after the oral hygiene instruction session; and.

T_4_ = 90 days after the oral hygiene instruction session.

The clinical outcomes were evaluated at baseline and at each follow‐up visit. Radiological outcome (MBL) was evaluated at baseline and 90 days after the instruction session (T_4_). The endoral radiographs were standardized using personalized silicone to maintain parallelism, axis, and position (Cosola et al., 2021).

### 2.5 Statistical analysis

The clinical parameters were expressed as mean ± standard deviation (SD). Student's *t*‐test for independent samples was used to evaluate the difference between the N and C groups. Differences were considered statistically significant at *p* ≤ 0.05. Statistical analyses were performed using Excel (Microsoft Windows 2020).

## 3 RESULTS

A total of 40 patients with a mean age of 49.8 ± 11.75 years completed the follow‐up sessions. The demographic data of both groups (PerioTabs® and chlorhexidine) are reported in Table 2. No significant differences were observed based on gender, age, or smoking habits (*p* > 0.05). A total of 70 dental implants were evaluated: 34 implants in the PerioTabs® group (test group) and 36 in the chlorexidine group (control group).

**Table 2:** Anamnestic data of two treatment groups of treatment, SD: Standard deviation, N: “Number of”

Treatment group	PerioTabs®	Chlorhexidine
*N* patients	20	20
Female	12	10
Male	8	10
Age (mean ± SD)	48.35 ± 11.75	51.25 ± 12.02
*N*° patients smoking <10 cigarettes	8	8
*N*° implants	34	36

### 3.1 Clinical assessment

The mean and standard deviation for each clinical parameter are reported in Table 3. All clinical outcomes significantly improved (*p* < 0 0.01) between T0 and T1 and between T1 and T2, except for PPD. There were significant differences between the groups in the gingival index (0–3) and FMBS (%) at T1, after the domiciliary use of both products. Patients in the PerioTabs® group showed the greatest benefit in FMBS (%) from full‐mouth NSPT when compared with patients assigned to the chlorhexidine group (*p* < 0.05) at T2. In addition, 19 patients in the PerioTabs® group showed signs of gingivitis at T0; this was reduced to five patients at T1, and none at T2. In the chlorhexidine group, 17 patients had gingivitis symptoms at T0, nine at T1, and two at T2. Therefore, it was not possible to analyze the recession. PPD did not show any differences between the groups and time points.

**Table 3:** Gingival index (mean value ± SD) mean pocket probing depth (PPD), FMBS (%) and respectively, *p*‐value, *p* < 0.05 in bold

Gingival index (0–3)	T_0_	T_1_	T_2_	T_3_	T_4_
PerioTabs® (*N* patients = 20)	2.30 ± 0.80	1.15 ± 0.75	0.05 ± 0.22	0.25 ± 0.44	0.45 ± 0.51
Chlorhexidine (*N* patients = 20)	2.15 ± 0.81	1.40 ± 0.94	0.25 ± 0.55	0.25 ± 0.55	0.60 ± 0.68
*P*‐value (*p* < 0.05 in bold)	*P = 0.560*	***P = 0.044***	*P = 0.260*	*P = 0.619*	*P = 0.354*
Mean Pocket Probing Depth (PPD)	T_0_	T_1_	T_2_	T_3_	T_4_
PerioTabs® (*N* patients = 20)	3.60 ± 1.27	2.80 ± 1.24	2.35 ± 0.93	2.35 ± 0.93	2.35 ± 0.74
Chlorhexidine (*N* patients = 20)	3.60 ± 1.39	3.10 ± 1.33	2.66 ± 1.00	2.67 ± 1.05	2.63 ± 0.91
P value	*P = 1.000*	*P = 0.084*	*P = 0.259*	*P = 0.256*	*P = 0.349*
FMBS (%)	T_0_	T_1_	T_2_	T_3_	T_4_
PerioTabs® (*N* patients = 20)	77.02 ± 20.39	**29.66 ± 15.23**	**7.91 ± 6.05**	15.40 ± 13.72	21.90 ± 15.65
Chlorhexidine (*N* patients = 20)	70.19 ± 17.31	**52.05 ± 14.78**	**17.58 ± 8.30**	21.58 ± 9.83	28.37 ± 10.83
*P*‐value (*p* < 0.05 in bold)		***P = 0.0001***	***P = 0.0002***	*P = 0.1096*	*P = 0.1386*

**Figure 1:**
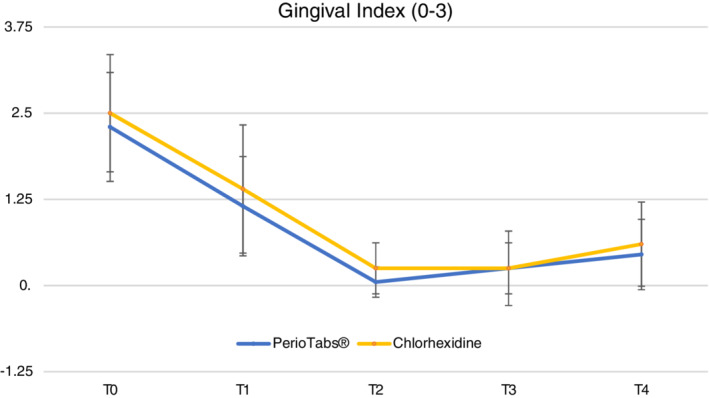
Gingival index (0–3) the GI drops down in both groups. After the domiciliary treatment (T1), the PerioTabs®‐group shows a significantly greater reduction in mean value relative to that of chlorhexidine

**Figure 2:**
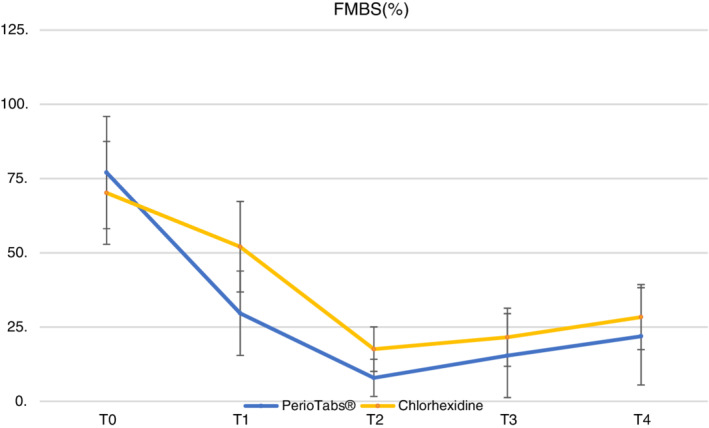
The full‐mouth bleeding scores (%) drop down in both groups, especially in T2 after the professional oral hygiene session. After the domiciliary treatment (T1), the PerioTabs® group shows a significant reduction in mean value relative to that of the chlorhexidine group

Figures 1 and 2 show the plots of the relative treatment effect for GI and reveal significant differences between the groups at T1. For FMBS, the figures show differences at T1 and T2. The graphics highlighted at T4 show a slightly greater worsening of these two parameters in the chlorhexidine group.

### 3.2 Implant sites

The mean and standard deviations for each clinical parameter for dental implants are shown in Table 4. A total of 70 dental implants were evaluated: 34 implants in the PerioTabs®‐group (test group) and 36 in the chlorexidine group (control group). Of the 34 implants evaluated in the PerioTabs® group, 31 were diagnosed with peri‐implant mucositis (five patients were smokers) and three with peri‐implantitis (three patients were smokers). In the chlorhexidine group, 34 implants showed peri‐implant mucositis and two implants showed peri‐implantitis in two different smoking patients. The main clinical outcomes of implants (mPI and mBI) significantly improved (*p* < 0 0.01) between T0 and T1 and between T1 and T2, except for PPD, which showed no differences in dental implants.

**Table 4:** Mean value ± SD of modified plaque index (0–3), modified bleeding index (0–3) and respectively, *p*‐value, *p* < 0.05 is in bold

Modified plaque index (0–3)	T_0_	T_1_	T_2_	T_3_	T_4_
PerioTabs® (*N* implants = 33)	2.54 ± 0.50	1.09 ± 0.58	0.06 ± 0.24	0.12 ± 0.33	0.48 ± 0.57
Chlorhexidine (*N* implants = 35)	2.41 ± 0.56	1.29 ± 0.80	0.26 ± 0.62	0.38 ± 0.65	0.71 ± 0.76
*P*‐value	*P = 0.308*	*P = 0.067*	***P = 0.048***	*P = 0.041*	*P = 0.087*
Modified Bleeding Index (0–3)	T_0_	T_1_	T_2_	T_3_	T_4_
PerioTabs® (*N* implants = 33)	2.36 ± 0.49	1.24 ± 0.56	0.21 ± 0.41	0.24 ± 0.43	0.30 ± 0.47
Chlorhexidine (*N* implants = 35)	2.29 ± 0.52	1.29 ± 0.67	0.29 ± 0.52	0.26 ± 0.44	0.40 ± 0.60
*P*‐value	*P = 0.526*	*P = 0.236*	*P = 0.343*	*P = 0.533*	*P = 0.329*

It was not possible to analyze MBL because no clinical differences were reported after treatment: less than 90 days was not sufficient to highlight significant differences between time points and between groups, and most patients had peri‐implant mucositis without any bone involvement. According to the new classification of periodontal and peri‐implant disease, after treatment, none of the implants with mucositis required peri‐implant surgery in either group during the study's observation period, because no signs of inflammation (bleeding or suppurations) and symptoms worsened. The five implants with peri‐implantitis (three of the 33 implants evaluated in the PerioTabs®‐group and two implants in the chlorhexidine group) did not present signs of suppuration, and the patients did not report pain; nevertheless, these five implants had no improvements in mBI at T_4_ compared with T_0_.

**Figure 3:**
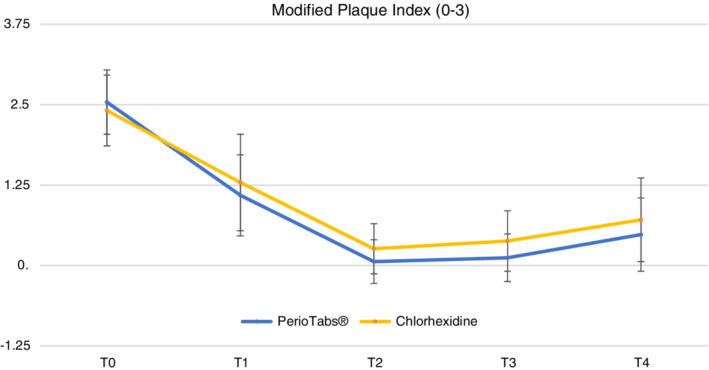
The modified plaque index (mPI, value between 0–3) drops down in both groups, especially in T2 after the professional oral hygiene session. In both groups the plaque grows after the same weeks in T3 and T4. No statistically significant differences are highlighted between the groups, with a *p*‐value <0.05, except for T2

There were significant differences between the groups in mPI (0–3) at T2, with the greatest benefit found in the PerioTabs® group compared with the chlorhexidine group (*p* < 0.05). Figure 3 demonstrates the trend of mPI, showing significant differences between each time point and T_0_.

**Figure 4:**
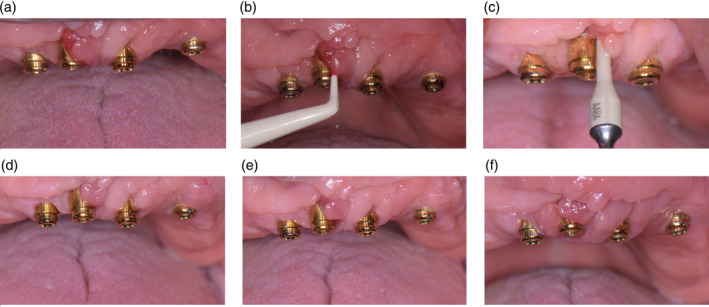
A patient in the PerioTabs® group that has four implants with peri‐implant mucositis, at each time‐point—A: T0, before dental check‐up; b: T0, during probing with a plastic tool; c: T1, clinical aspect of the mucosa around the dental implants after 1 week of domiciliary PerioTabs® therapy, during the full‐mouth NSPT; d: T2, during the check‐up 3 days after the FMD; e: T3, during the check‐up; f: T4, during the check‐up

Figure 4 reports the clinical aspect of the only edentulous patient within the PerioTabs®‐group with four implants. At each time point, a clear improvement in gingival health was observed.

### 3.3 Patients' reported outcomes

In addition, patient outcomes (VAS and OHrQoL) significantly improved after both types of nonsurgical maintenance protocols (*p* < 0.001), as reported in Table 5. There were no significant differences between the groups in VAS scale and OHIP‐14‐score (OHrQoL), but patients in the PerioTabs® group showed a slightly better trend after treatment. No signs of severe side effects or allergic reactions were observed in either group. Nevertheless, two patients in the chlorhexidine group reported burning sensations, and one patient had severe upper palatal tooth staining.

**Table 5:** VAS (0–10) and OHrQoL (OHIP‐14) with respectively, *p*‐values, no values were significant with *p* < 0.05

VAS on implants (0–10)	T_0_	T_1_	T_2_	T_3_	T_4_
PerioTabs® (*N* patients = 20)	3.72 ± 1.75	1.94 ± 1.27	0.30 ± 0.58	0.18 ± 0.39	0.18 ± 0.39
Chlorhexidine (*N* patients = 20)	3.6 ± 2.00	1.91 ± 1.36	0.34 ± 0.59	0.29 ± 0.52	0.26 ± 0.50
*P*‐value (*p* < 0.05 in bold)	*P = 0.782*	*P = 0.937*	*P = 0.781*	*P = 0.357*	*P = 0.496*
OHrQoL (OHIP‐14)	T_0_	T_1_			
PerioTabs® (*N* patients = 20)	2.59 ± 0.48	0.71 ± 0.39			
Chlorhexidine (*N* patients = 20)	2.61 ± 0.58	0.90 ± 0.31			
*P*‐value (*p* < 0.05 in bold)	*P = 0.924*	*P = 0.113*			

## 4 DISCUSSION

The results of the present study confirmed the efficacy of MFMD as a key treatment for periodontal and peri‐implant disorders. After 1 week of domestic intervention, the patients managed to reduce the amount of localized plaque, pain, and inflammation, while improving their overall oral health condition. Furthermore, all clinical parameters significantly improved after only 1 week of domestic care (T1), and the trend continued up to T2 (3 days after NSPT).

The probing depth did not change significantly between the groups at the implant level. These results are consistent with the limitations of this clinical parameter when used in peri‐implant mucositis diagnosis. In fact, PPD does not provide any information about peri‐implant health because of the variability in implant and prosthesis type, and their positioning and use of different prosthetic components (Coli & Sennerby, 2019).

In addition, the clinical parameters around the dental implants (mPI and mBI) significantly improved after only 1 week (T1) and 3 days after the operative session (T2), and no signs of inflammation or pain were reported after treatment. These results indicated that peri‐implant mucositis was stabilized, and no implants needed adjunctive surgery after motivation, domestic treatment, and supportive non‐surgical therapy. However, all the implants diagnosed with peri‐implantitis at baseline still presented with bleeding upon probing at the last follow‐up visit. This is because frank peri‐implantitis is a multifactorial disorder often influenced by systemic conditions, implant mal‐positioning, and the type/quality of the prosthetic restoration.

The group using PerioTabs® showed better clinical outcomes with respect to GI (0–3) at T1 compared with patients assigned to the use of chlorhexidine paste and mouth rinse. Patients in the PerioTabs® group also showed a greater reduction in FMBS after professional treatment than those in the chlorhexidine group. Other clinical data showed no statistically significant differences between the groups. These findings may be due to the preliminary reduction in local inflammation and plaque formation achieved with MFMD therapy. The main findings of the present study are in line with those published by Marconcini et al., (2019), who showed that the effectiveness of domestic patient care is directly related to the clinical improvement observed in patients before the NSPT (Marconcini et al., 2019).

The crossover study of Sekino et al. (2004) highlighted that a chlorhexidine mouthwash before surgery could significantly decrease the salivary and tissue bacterial counts and delay plaque formation (Sekino et al., 2004). The substantivity of a 0.2% chlorhexidine mouthwash can have an antibacterial effect for up to 3 h. Subsequently, the microbiota spontaneously recolonized the oral cavity to the baseline level (immediately after the NSPT) (Quintas et al., 2015). The trends seen in the GI and MPI in the present study at T3 and T4 confirm these data and reinforce the notion that the specific individual characteristics of the microbiota could return to the same situation as seen before the stimulus (local antimicrobial substance) was applied, and could even worsen with a major shift in the salivary composition, leading to more acidic conditions and a lower nitrite availability, especially among healthy individuals (Bescos et al., 2020; García‐Caballero et al., 2013).

Regarding patient‐related endpoints, all patients reported a significant improvement in all outcomes. The pain measured by VAS was improved at seven and 10 days, and the seven‐day OHrQoL also showed improvement (Cosola et al., 2018), allowing us to conclude that the benefits of the domestic oral care instructions and the patients' motivation did not only result in an improved anti‐plaque effect, but also in their increased well‐being (Meza‐Mauricio et al., 2018).

The MFMD protocol supports the modern tendency towards a more patient‐centered ‘self‐care’ approach, whereby the patient takes increased responsibility for his/her own therapy (Marconcini et al., 2019). A oral hygiene domestic regimen is recommended for the first seven to 15 days; then, the motivation of the patient needs to be reinforced, and the NSPT is performed at this stage (Genovesi et al., 2014). In this initial seven‐ to 15‐day antimicrobial “shock” therapy, chlorhexidine, particularly used as a mouth rinse, represents the gold standard agent (Zhao et al., 2020). However, since chlorhexidine often induces side effects such as oral mucosa desquamation, tooth staining, and alterations in taste, alternatives might be evaluated. Our study showed that PerioTabs® can be a valid alternative as an effective initial domestic therapy for MFMD (D'Ercole et al., 2017; Slot et al., 2014).

In this study, no non‐desired side effects of PerioTabs® were observed, whereas two patients in the chlorhexidine group reported burning sensations after the use of chlorhexidine for 10 days. One patient also presented with severe staining of the palatal surfaces of the upper teeth. In addition, although daily preparation of the PerioTabs® brushing solution by the patient at home required some time and could be considered less consumer‐friendly than the use of a simple toothpaste, the patients regarded PerioTabs® as an actual drug to be taken seriously, and consequently, were highly motivated to follow precisely the directions for use (Coenye et al., 2008; Goguta et al., 2021). In addition, patients preferred the one‐step approach of brushing with the PerioTabs® solution over the two‐step approach of chlorhexidine toothpaste and mouthwash.

A common limitation of both PerioTabs® and chlorhexidine is that they should not be used as long‐term preventive therapies. However, according to the manufacturer, PerioTabs® can be used safely every 90 days as a biofilm removal maintenance procedure. A limitation of this study is that local inflammation and plaque accumulation for each time point were only measured indirectly. It would be interesting to calculate the bacterial load through bacterial plaque analysis or to check for pro‐inflammatory markers such as C‐reactive protein (CRP) at each time point. Furthermore, pain and improvement of oral health were self‐reported in this study, and taking personal views about one's own health may not be a reliable, objective method. Additional studies with a larger sample of patients and their stratification according to peri‐implant disease severity are needed to confirm the present findings.

Aerosols represent a major challenge in dental settings, for which a pre‐procedural rinse with an antiseptic mouthwash such as chlorhexidine has been proposed (D'Ercole et al., 2017). Therefore, given the properties of the NitrAdine®, future studies aimed at assessing airborne contamination after dental treatment of patients who have had a pre‐operative rinse of PerioTabs® are recommended (Coenye et al., 2008; Glass et al., 2004). It is also unknown whether the level of gingival inflammation contributes to the bacterial load within aerosol contamination. In this respect, MFMD may offer an interesting approach. Reducing the patient's plaque and inflammation by means of a robust domestic dental hygiene protocol prior to professional treatment could enable clinicians to work more safely with a low level of airborne contamination and a lower production of contaminated aerosol during periodontal debridement.

The findings of the present study indicate that there are alternatives to chlorhexidine for the management of peri‐implant mucositis by NSPT. Specifically, PerioTabs® was found to be associated with reduced pain and dental anxiety, and greater product acceptance, with fewer side effects than chlorhexidine.

## CONFLICT OF INTEREST

None declared. Bonyf AG provided all the materials to support the professional and domiciliary treatment of the patients.

## AUTHORS CONTRIBUTION

Conceptualization, A.G. and S.M.; methodology, S.C.; software, G.O.; validation, S.C., and U.C.; formal analysis, E.G.; investigation, G. O.; resources, S.M. and U.C.; data curation, A.G. and G.O.; writing‐original draft preparation, S.C. and S.M..; writing‐review and editing, S.C. and E.G..; visualization, E.G.; supervision, S.M.; project administration, U.C.; funding acquisition, U.C. All authors have read and agreed to the published version of the manuscript.

## Data Availability

The data that support the findings of this study are available on request from the corresponding author. The data are not publicly available due to privacy or ethical restrictions.
